# SURGICAL TREATMENT OF ACROMIOCLAVICULAR DISLOCATION: HOOK PLATE VERSUS SUTURE BUTTON

**DOI:** 10.1590/1413-785220233101e252916

**Published:** 2023-04-17

**Authors:** Muhsin Dursun, Guray Altun, Murat Ozsahin

**Affiliations:** 1 EPC Special Hospital, Department of Orthopedics and Traumatology, Adana, Turkey.; 2 University of Health Sciences Umraniye Training and Research Hospital, Department of Orthopedics and Traumatology, Istanbul, Turkey.; 3 Middle East Special Hospital, Department of Orthopedics and Traumatology, Adana, Turkey.

**Keywords:** Acromioclavicular Joint, Surgical Procedures, Clavicle, Joint Dislocations, Articulação Acromioclavicular, Procedimentos Cirúrgicos, Clavicula, Luxações Articulares

## Abstract

**Objectives:**

We aimed to compare the functional and radiographical outcomes of reconstruction of acute unstable acromioclavicular joint (ACJ) dislocation using Hook Plate (HP) versus Suture Endobutton (SE) fixation techniques.

**Methods:**

Forty-six consecutive patients with grade III to V ACJ dislocation according to Rockwood classification who underwent either HP or SE fixation in the period between January 2017 and June 2020 were evaluated. The treatment modalities were divided into either HP or SE fixation. The radiological assessment included standard anterior-posterior (AP) views to evaluate coracoclavicular (CC) distances for vertical reduction.

**Results:**

CC distances were grouped as preoperative (CC^1^), early postoperative (CC^2^), and late postoperative (CC^3^). The distance variance between CC^2^ and CC^3^ was referred as ΔCC (CC^3^ - CC^2^). A statistically significant difference was found in ΔCC between the two groups (p=0.008). ΔCC was significantly higher in the SE group compared to the HP group (p<0.05). The Constant and UCLA Scores of patients in the SE group were found to be significantly higher than in the HP group patients.

**Conclusion:**

Clinical outcomes were more satisfactory in patients with acute unstable ACJ dislocation who underwent SE compared to HP procedures, at the end of the first year. *Evidence Level IV; Case Series*.

## RESUMO

**Objetivo:**

Nosso objetivo foi comparar os resultados funcionais e radiográficos da reconstrução da luxação instável aguda da articulação acromioclavicular (ACJ) utilizando técnicas de fixação com placa com gancho (HP) versus botão de sutura (SE).

**Métodos:**

46 pacientes com luxação da ACJ de grau III a V, de acordo com a classificação de Rockwood, que foram submetidos à fixação com HP ou SE no período de janeiro de 2017 a junho de 2020, foram avaliados. As modalidades de tratamento foram divididas em fixação HP ou SE. Na avaliação radiológica, foi utilizada a incidência antero-posterior (AP) para avaliação da redução vertical, por meio da medida da distância córaco-clavicular (CC).

**Resultados:**

As distâncias CC foram agrupadas em pré-operatória (CC^1^), pós-operatória imediata (CC^2^) e pós-operatória tardia (CC^3^). A variação da distância entre (CC^2^) e (CC^3^) foi denominada ΔCC. Uma diferença estatisticamente significativa foi encontrada na ΔCC entre os dois grupos (p=0,008). O ΔCC foi significativamente maior no grupo SE em comparação com o grupo HP (p <0.05). As pontuações de Constant e UCLA dos pacientes do grupo SE foram significativamente mais elevadas do que as dos pacientes do grupo HP.

**Conclusão:**

Os resultados clínicos foram mais satisfatórios com a técnica SE em comparação com a HP ao final do primeiro ano. ***Nível de Evidência IV; Série de casos.***

## INTRODUCTION

Acromioclavicular joint injuries are approximately 12 % of all shoulder injuries and most commonly occurs by a direct force to the acromion under an adducted arm.^1,2^ The ACJ is an important structure connecting the axial skeleton to the upper extremity, the upper extremities being suspended by strong coracoclavicular (CC) ligaments and an acromioclavicular (AC) ligament. Thus, dislocation with torn AC and CC ligaments often leads to severe functional impairment of the injured shoulder. Due to the limited healing potential of the CC ligament, appropriate treatment is necessary in the acute phase.^3^ Treatment of ACJ dislocations remains a controversial topic. There is a general consensus in the literature to treat acute Rockwood grade I and II injuries conservatively and grade IV, V and VI injuries operatively.^4^ However, treatment of acute grade III injuries remains controversial.^4,5^ Early surgical repair of grade III ACJ dislocations results in better outcomes and an earlier return to sport activities, although conservative treatment is also recommended.^6-8^ Multiple surgical options exist, including CC screws, hook plates (HP), suture endobutton (SE) CC fixations, and anatomic ligament reconstructions with tendon grafts, but none can be regarded as the gold standard treatment.^9^ The HP fixation technique is an effective treatment option for ACJ dislocation and is widely used owing to the simplicity of the surgical technique along with good clinical outcomes.^10,11^ It also has the advantage of attaining reduction in the horizontal and vertical planes.^12^ However, several documented complications have been reported, such as shoulder impingement, rotator cuff lesion, infection and bony erosion.^13^ Eventually improved implants that are less invasive have been developed. These include the SE technique, which consists of a suspensory fixation device between the clavicle and the coracoid process that can stabilize the ACJ and reinforce the CC ligaments. Numerous studies have shown favorable clinical and radiographic results from this procedure.^13^ In the literature, several comparative studies have aimed to evaluate treatment outcomes for ACJ dislocation with HP versus SE fixation techniques.^13-18^ The clinical results of both surgical techniques have been satisfactory, although there is no clear consensus on which method produces superior outcomes. In the present study, we aimed to compare the functional and radiographical outcomes of reconstruction of acute unstable ACJ dislocation using HP versus SE fixation techniques.

## MATERIAL AND METHODS

All procedures performed in this study involving human participants were in accordance with the ethical standards of the institutional and national research committee and with the 1964 Helsinki Declaration and its later amendments or comparable ethical standards. Informed consent was obtained from each participant included in the study. The study was approved by the Ethics Committee of the same hospital (Decision no: 228 dated: 03.03.2021). No approval from the National Ethics Committee was necessary as it was a non-interventional observational study.

104 consecutive patients with grade III to V ACJ dislocation (according to Rockwood classification measured with true AP projection) who underwent either HP or SE fixation at our clinic in the period between January 2017 and June 2020 were compared and evaluated retrospectively. The inclusion criteria for the study were as follows: (1) no history of shoulder injuries or related surgeries, (2) acute ACJ dislocation (<2 weeks after trauma) of Rockwood type III or higher (3) A follow-up period of at least 12 months. The following patients were excluded: (1) open or chronic dislocations, (2) dislocations combined with neurovascular or vital organ injury, and (3) ipsilateral upper limb fractures and/or dislocations. The treatment modalities were divided into either HP or SE fixation and all surgeries were performed by two senior surgeons. Each surgeon performed only HP or SE technique. Sixty-two patients were treated using HP fixation while 42 patients were treated with the SE technique. Of the 104 patients initially screened, 58 met one or more of the exclusion criteria or did not meet inclusion criteria; therefore, the remaining 46 patients (23 HP and 23 SE) were included in the final study group. The surgical method was decided according to surgeon's preference and experience.

Demographic and clinical data regarding age, sex, hand dominance, mechanism of injury, time from trauma to surgery, time from surgery to the return to daily activities, and length of follow-up were collected. A functional assessment was performed by two independent reviewers using the Constant score and University of California Los Angeles (UCLA) score. Radiological assessment included standard anterior-posterior (AP) views to evaluate CC distances for vertical reduction. The CC distance was defined as the vertical distance between the anterior–inferior border of the clavicle and the superior border of the coracoid process. All measurements were performed and analyzed in three stages: preoperatively, in the early postoperative period, and at the time of the final follow-up. The affected ACJ was also evaluated for any signs of postoperative degenerative arthritis, loss of reduction, osteolysis and acromio-coracoclavicular ligament ossification. CC distances were grouped as preoperative (CC^1^), early postoperative (CC^2^) and late postoperative (CC^3^). The distance variance between CC^2^ and CC^3^ was referred as ΔCC (CC^3^ - CC^2^). Two criteria for radiological failure were identified in the current study. The first one was comparison of the CC distance measured immediately after the surgery and at the final follow-up. The second was comparison of CC distances measured on the operated side and on the unaffected side at the final follow-up.

### Surgical Technique:

**Figure 1:**
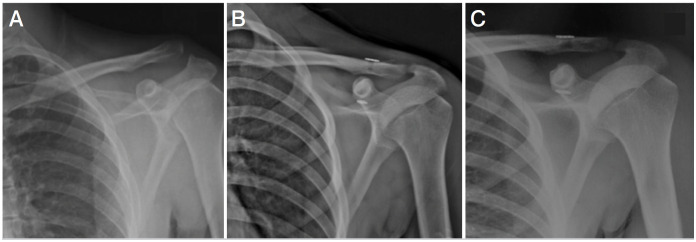
A) Preoperative left shoulder XR of 47 years old male patient with Type 3 acromioclavicular dislocation B) Early postoperative XR of patient who underwent suture endobutton (SE). An overcorrection of the coracoclavicular distance can be observed. C) Postoperative XR of the same patient at the end of a one-year follow up. Resolution of the overcorrection of coracoclavicular distance can be observed.

**Figure 2:**
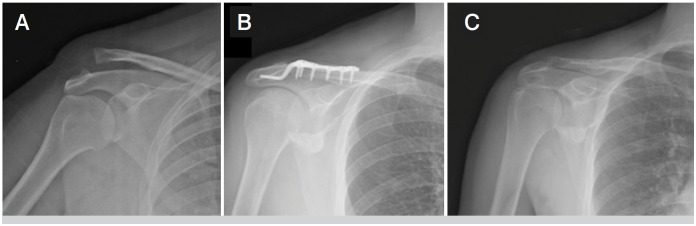
a) Preoperative right shoulder XR of 42 years old male patient with type 5 acromioclavicular dislocation b) Early postoperative XR of the patient who underwent hook plate (HP) c) Postoperative XR of the same patient after HP removal.

Patients were placed in the beach chair position under general anesthesia. The upper extremity was prepared and draped in the usual sterile manner, and appropriate antibiotic prophylaxis was administered before the incision. An approximately 6 cm incision was made at the superior end of the injured ACJ. For HP fixation, the patient's soft tissues were dissected until the ACJ became visible. Next, the anterior and posterior edges of the acromion were located, and their midpoint was marked to guide the placement of the plate. The ACJ dislocation was reduced, and a hook plate was placed over the ACJ. (Figure 1) The hook was placed as posteriorly as feasible to ensure complete attachment to the acromion and to avoid subacromial impingement (on the supraspinatus bursa or rotator cuff). For SE fixation, a 5 cm incision was made at the top of the clavicle, 2 cm medial to ACJ. The pectoral muscle was dissected out from clavicle and meticulous dissection was performed down to the base of the coracoid process. Under C-arm X-ray machine visualization, the bony tunnels to the clavicle and coracoid process were drilled during separate steps. First, a 2.4-mm guide pin was inserted in a cephalad to caudal direction at the base of the coracoid process. The guide pin was aimed at the center of the coracoid process and close to the neck. A 4.0-mm cannulated drill was used, and care was taken to avoid advancing the guide pin while drilling. Then, a bony tunnel was drilled in a similar manner at the center of the distance between the anterior and posterior borders of the clavicle. The guide wire and drill were removed; the suture button was inserted through the clavicle, and then through the coracoid tunnel using the button inserter. The oblong button was flipped and seated underneath the coracoid process using a pusher. Finally, the ACJ was reduced and placed in the anatomical position under fluoroscopic visualization, and the round button was advanced to the cephalad surface of the clavicle. (Figure 2) The subcutaneous tissues and skin were closed in the usual manner. After surgery, a standard rehabilitation program was applied to all patients, with the use of a shoulder immobilizer sling for 4 weeks. The patients were allowed to start gentle pendulum & Codman's exercises and perform elbow flexion & extension exercises as tolerated postoperatively. In the fourth week, the arm sling was removed, and stretching exercises were conducted to increase the range of motion; while strengthening exercises were started after 8 weeks. Standard exercises in a home exercise program were recommended. Full active movement was allowed at 6 weeks and a return to manual work was allowed at 2 months. Contact sports were not allowed until 6 months postoperatively.

### Statistical Analysis

Statistical analyses were carried out with IBM SPSS (Statistical Package for the Social Sciences for Windows, version 21.0, Armonk, NY, IBM Corp.). Frequency tables and descriptive statistics were used to interpret the findings. Parametric methods were used for measurement values suitable for normal distribution. Shapiro-Wilk test was used for measurement of normal distribution. In accordance with the parametric methods, the “Independent Sample-t” test (t-table value) method was used to compare the measurement values of the two independent groups. Nonparametric methods were used for measurement values that were not suitable for normal distribution. In accordance with nonparametric methods, “Mann-Whitney U” test (Z-table value) method was used to compare the measurement values of two independent groups. The “Friedman” test (χ^2^-table value) method was used to compare the measurement values of three or more dependent groups. Dunn correction was applied for paired comparisons of variables that differed significantly for three or more groups. “Spearman” correlation coefficient was used to examine the relationship between two quantitative variables that did not have a normal distribution. “Pearson-χ^2^” and continuity correction cross tables were used to examine the relationship between two qualitative variables. For each measurement, the interclass correlation coefficient (ICC) and 95% confidence interval were reported. Correlation was classified as poor (0.00–0.20), fair (0.21–0.40), moderate (0.41–0.60), good (0.61–0.80), or excellent (0.81–1.00).

## RESULTS

All patients had an ACJ dislocation and underwent fixation with either HP or SE technique. The average age of the SE group was 38.17 ± 12.34 years and the HP group was 48.69 ± 13.55 years. No statistically significant differences in age, sex, affected side, dominant limb, trauma mechanism or distribution of Rockwood classification were found between the two groups (p>0.05). The groups were found to be independent and homogeneous for the specified characteristics. Detailed data are presented in Table I. There was no statistically significant difference in CC^1^, CC^2^ or CC^3^ distances between the groups (p>0.05). A statistically significant difference was found in ΔCC (Z=-2.652; p=0.008) between the two groups. ΔCC was significantly higher in the SE group compared to the HP group (p <0.05) (Table II). ICC of the observers for the radiological measurements was 0,89 which showed excellent reliability. The Constant and UCLA Scores of patients in the SE group were 97.65±2.87 and 38.70±1.30 and for the HP group they were 94.59±3.06 and 34.14±1.73, respectively. Clinical scores of patients in the SE group were found to be significantly higher than those of the patients in the HP group (Z=-3.495; p=0.000, Z=-5.718; p=0.000, for SE and HP, respectively) (Table III). A modest but statistically significant correlation was found between ΔCC differences and the Constant or UCLA Score in the SE group (p <0.05). As the Constant or UCLA scores increased, the ΔCCshowed a decrease. Likewise, as the Constant and UCLA scores decreased, the ΔCC value increased. No statistically significant correlation could be identified between ΔCC and the Constant and UCLA Scores of the HP group (p> 0.05) (Table IV).

**Table 1:** Characteristics of forty-six patients who underwent fixation of acromioclavicular joint dislocation with hook plate (HP) or suture endobutton (SE).

Group	SE (n=23)		HP (n=23)		Statistical analysis* Probability
Variable	n	%	n	%	
**Age groups**					
<30	8	34.9	8	34.8	χ^2^=0.397 p=0.941
30-39	5	21.7	3	13.0	
40-49	5	21.7	6	26.1	
≥50	5	21.7	6	26.1	
**Gender**					
Male	18	78.3	16	69.0	χ^2^=0.190 p=0.663
Female	5	21.7	7	31.0	
**Limb**					
Right	13	56.5	14	60.1	χ^2^=0.023 p=0.879
Left	10	43.5	9	39.9	
**Dominant limb**					
Right	22	95.7	17	73.9	χ^2^=1.705 p=0.192
Left	1	4.3	6	26.1	
**Trauma**					
Sport	7	30.4	6	26.1	χ^2^=0.424 p=0.935
Simple fall	10	43.5	10	43.5	
Bicycle accident	4	17.4	5	21.8	
Fall from height	2	8.7	2	8.6	
**Rockwood status**					
Type 3	9	39.1	6	26.1	χ^2^=0.341 p=0.559
Type 5	14	60.9	17	73.1	

* ”Pearson-χ^2^” and continuity correction cross tables were used to examine the relationship between two qualitative variables.

**Table 2:** Comparison of CC distances of patients who underwent fixation of acromioclavicular joint dislocation with either hook plate (HP) or suture endobutton (SE).

Group Variables	SE (n=23)		HP (n=23)		Statistical analysis* Probability
	X S.S.(cm)	Median [IQR]	X S.S.(cm)	Median [IQR]
*∆*CC	0.82 0.70	0.7 [0.4]	0.45 0.30	0.4 [0.5]	Z=-2.652 p=0.008
CC distance					
CC^1^	21.23 4.24	23.4 [8.2]	21.91 3.64	22.8 [5.4]	Z=-0.194 p=0.846
CC^2^	10.71 1.10	10.7 [1.3]	10.42 1.37	10.2 [2.1]	Z=-1.230 p=0.219
C^C^3	11.53 1.32	11.5 [1.8]	10.87 1.45	10.8 [2.1]	t=1.681 p=0.099
Statistical analysis Probability	χ^2^=46.000 p=0.000 [1-2.3] [2-3]		χ^2^=56.214 p=0.000 [1-2.3] [2-3]		

* ”Independent Sample-t” test (t-table value) statistics was used to compare the measurement values of two independent groups with normal distribution. “Mann-Whitney U” test (Z-table value) was used for the comparison of measurement values of two independent groups that did not have normal distribution; “Friedman” test (*χ^2^*-table value) statistics were used to compare three or more dependent groups.

**Table 3:** Comparison of parameters according to the groups.

Group	Endobutton (n=23)		Hook Plate (n=23)		Statistical analysis* Probability
Variable		Median [IQR]		Median [IQR]	
Follow-up time	19.70±4.85	19 [8.0]	26.07±9.15	25 [17.0]	Z=-2.261 p=0.024
Constant score	97.65±2.87	98 [4.0]	94.59±3.06	95 [4.0]	Z=-3.495 p=0.000
UCLA score	38.70±1.30	39 [2.0]	34.14±1.73	34 [2.0]	Z=-5.718 p=0.000

* ”Mann-Whitney U” test (Z-table value) statistics were used in comparing the measurement values. Of two independent groups with no normal distribution.

**Table 4:** Correlation analyses between ΔCCand two different functional scores of patients who underwent fixation of acromioclavicular joint dislocation with hook plate (HP) or suture endobutton (SE).

	∆CC differences			
Group	SE (n=23)		HP (n=23)	
	r	p	r	p
Constant score	-0.438	0.036	-0.272	0.153
UCLA score	-0.445	0.033	0.047	0.809

* Spearman correlation coefficient was used to examine the relationship between two quantitative variables that did not have normal distribution.

## DISCUSSION

In the current study, the radiological and clinical scores of patients with ACJ dislocation who underwent either HP or SE fixation were evaluated. A statistically significant increase in the CC distance was identified in patients who underwent SE fixation. However, at the final follow up after at least 12 months, the UCLA and Constant scores were lower in the HP group.

HP fixation is a dynamic technique for the surgical fixation of ACJ dislocation. It works on the principle of generating a leverage arm through the proximal end of the plate to the acromion as a dynamic fixation and can hold the position of the clavicle and CC distance stable. This technique can be used with satisfactory results to treat acute injuries and may be combined with ligament reconstruction for chronic injuries as well. However open reduction and internal fixation with HP may cause postoperative complications such as subacromial osteolysis, rotator cuff rupture, acromial fracture or impingement syndrome.^16-19^ Furthermore, the fact that HP causes limitation and pain in shoulder movements after a period of time entails the need to remove it with a second surgery. This has negative effects on the rehabilitation process and the eventual clinical outcome. In the cases examined in the current study, acromial osteolysis was detected in only two patients and the implant was removed at the seventh and eighth months. The mean duration of postoperative time up to implant removal for the rest of the patients was six months. We attribute the low complication rate of this study to the short duration of retention of the implant with the HP technique. In the literature, the complications of SE technique include loss of reduction, coracoid process fracture, implant failure and overcorrection. In the cases included in the current study, overcorrection was detected in only one patient and the final outcome according to the clinical scores at the follow-up period was excellent (Constant: 100, UCLA: 40)

The SE technique has been reported to be applied not only with two endobuttons, one for clavicle and one for coracoid separately. Furthermore, different fixation technics were described as, Twin Tail Tight Rope ^®^, with two endobuttons for the clavicle and one button for coracoid; Double Tight Rope^®^ two endobuttons for the clavicle and two buttons for coracoid.^4,20^ These enhanced techniques can provide horizontal stability with the fiber wire in the ACJ.^4^ However, with these non-physiological fixation methods, increased stiffness can cause implant failure in coracoid fixation (20). Nonetheless, in comparative studies, vertical and horizontal stability of the double endobutton technique was described to be better than the single system; however, there was no significant difference in the clinical scores and the CC distance.^21^ In the current study, the increase in CC distance at the one-year follow up did not have a negative effect on clinical scores in the SE group as well.

In a meta-analysis, Weihui et al. suggested that the SE technique showed better outcomes compared to the HP technique in functional recovery and pain. The same study showed that when the CC distances were evaluated radiologically, an acceptable reduction loss was observed with the SE technique.^22^ In another meta-analysis, Wang et al. reported that the SE technique showed functionally better results compared to the HP technique; however no statistically significant difference in CC distance and complications could be identified.^13^ In the current study, we identified a statistically significant increase in CC distance between the first measurement at the early-postoperative period and the next measurement at the first-year follow-up in patients treated with the SE method compared to the patients treated with the HP method. But a modest significant correlation was found between clinical scores and ΔCC differences in SE group. This may have resulted from the development of complications in the HP patients despite the removal of the plate after a short time. Although both SE and HP are dynamic techniques, the support point of HP is the subacromial face of the acromion and it was shown that horizontal and vertical plate movements in this area cause mechanical trauma in both inferior border of the acromion in the superior area and bursal side of the rotator cuff in the inferior area.^19^

A direct relationship between the ΔCC and Constant/UCLA scores was detected in the SE group. These parameters have a reciprocal relationship with each other. Of note, no statistically significant relationship was identified between ΔCC and clinical scores in the HP group. However, the lack of a statistically significant relationship does not necessarily lead to an interpretation of no relationship between ΔCC and clinical satisfaction in the HP group. In fact, the change in ΔCC in the HP group was very small, which precluded the establishment of a relationship between ΔCC and clinical scores. Rather, the bigger change in ΔCC in patients in the SE group allowed the establishment of relationship with clinical scores that reached statistical significance.

A cost analysis of both surgical techniques in our health care system indicated that the implant and material costs of both HP and SE techniques were comparable. However, the need for a second surgery with the use of HP enhanced the overall costs. These costs may differ from region to region and with health policies; nonetheless, the decision for use of either technique during surgery may be influenced by the prevalent economic conditions and secondary surgical risk factors relevant to the patient's health.

The limitations of the study that need to be considered are as follows: The current study is not the first one to compare the outcomes of use of either of SE or HP techniques, there are several studies in the published literature that have reported such comparisons. However, technical improvements such as fixation material technology and surgical choices have been updated during the last decade. Furthermore, recent developments may affect clinical scores and radiological outcomes. Secondly, horizontal stability has not been assessed for either group of patients in the current study. Recent studies have indicated that vertical stability is more significant in affecting clinical scores than horizontal stability; in addition, too much stiffness of implant fixation may result in failure.

## CONCLUSION

Both SE and HP techniques offered beneficial outcomes in relieving pain of dislocation and improving function of ACJ at the end of one-year follow-up in the postoperative period. A significant increase in CC distance was detected in the SE group at the end of the first year compared to the HP group; clinical outcomes were also more satisfactory with SE compared to HP.
